# Motion‐compensated gradient waveforms for tensor‐valued diffusion encoding by constrained numerical optimization

**DOI:** 10.1002/mrm.28551

**Published:** 2020-10-13

**Authors:** Filip Szczepankiewicz, Jens Sjölund, Erica Dall’Armellina, Sven Plein, Jürgen E. Schneider, Irvin Teh, Carl‐Fredrik Westin

**Affiliations:** ^1^ Harvard Medical School Boston Massachusetts USA; ^2^ Radiology Brigham and Women’s Hospital Boston Massachusetts USA; ^3^ Diagnostic Radiology, Clinical Sciences Lund Lund University Lund Sweden; ^4^ Elekta Instrument AB Stockholm Sweden; ^5^ Department of Information Technology Uppsala University Uppsala Sweden; ^6^ Leeds Institute of Cardiovascular and Metabolic Medicine University of Leeds Leeds United Kingdom

**Keywords:** diffusion magnetic resonance imaging, gradient waveform design, motion and flow compensation, tensor‐valued diffusion encoding

## Abstract

**Purpose:**

Diffusion‐weighted MRI is sensitive to incoherent tissue motion, which may confound the measured signal and subsequent analysis. We propose a “motion‐compensated” gradient waveform design for tensor‐valued diffusion encoding that negates the effects bulk motion and incoherent motion in the ballistic regime.

**Methods:**

Motion compensation was achieved by constraining the magnitude of gradient waveform moment vectors. The constraint was incorporated into a numerical optimization framework, along with existing constraints that account for b‐tensor shape, hardware restrictions, and concomitant field gradients. We evaluated the efficacy of encoding and motion compensation in simulations, and we demonstrated the approach by linear and planar b‐tensor encoding in a healthy heart in vivo.

**Results:**

The optimization framework produced asymmetric motion‐compensated waveforms that yielded b‐tensors of arbitrary shape with improved efficiency compared with previous designs for tensor‐valued encoding, and equivalent efficiency to previous designs for linear (conventional) encoding. Technical feasibility was demonstrated in the heart in vivo, showing vastly improved data quality when using motion compensation. The optimization framework is available online in open source.

**Conclusion:**

Our gradient waveform design is both more flexible and efficient than previous methods, facilitating tensor‐valued diffusion encoding in tissues in which motion would otherwise confound the signal. The proposed design exploits asymmetric encoding times, a single refocusing pulse or multiple refocusing pulses, and integrates compensation for concomitant gradient effects throughout the imaging volume.

## 1 INTRODUCTION

Tissue movement during diffusion encoding can lead to phase dispersion that is erroneously attributed to diffusion or cause gross signal dropout. For example, the relatively slow and incoherent movement of blood in capillaries has a measurable impact on the diffusion‐weighted signal at low b‐values and carries information about the vasculature and can be mistaken for fast diffusion, or so called “pseudo diffusion.”^1^ Other sources of motion include cardiac and pulmonary motion. These influence diffusion measurements in the brain by arterial pulsation^2^, ^3^ and by gross movement of tissue, such as in chest, cardiac and kidney imaging,^4^, ^5^, ^6^ or from vibrations induced by the diffusion encoding itself.^7^, ^8^, ^9^

To combat this artifact, diffusion‐encoding gradient waveforms have been designed to be “motion‐compensated”^4^, ^10^ (ie, modulated in a way that introduces no phase shift in spin that move without changing direction during the encoding). Efforts have previously covered conventional diffusion encoding along a single direction at a time, here referred to as “linear b‐tensor encoding.” For example, constant gradients with multiple refocusing pulses have been used to yield velocity compensation, and bipolar gradient waveforms have been used for the same purpose.^11^, ^12^, ^13^, ^14^, ^15^ More recently, Aliotta et al^16^ developed a flexible optimization framework to tailor motion‐compensated waveforms to arbitrary encoding times, and Peña‐Nogales et al^17^ used a similar approach to also include compensation for concomitant gradients.^18^

In addition to conventional linear b‐tensor encoding, it has been shown that complementary information about tissue microstructure can be extracted using diffusion encoding along more than one direction per signal readout.^19^, ^20^ In this work, we describe such “multidimensional” encoding with a b‐tensor^21^ (because it cannot be described merely by a direction and b‐value), and therefore refer to the diffusion encoding as “tensor‐valued.”^22^ Because the diffusion encoding b‐tensor can have varying encoding strength along different directions, the b‐tensor can be said to have a “shape.” By modulating the b‐tensor shape, the effect of microscopic diffusion anisotropy can be teased out, facilitating the quantification of parameters that are not accessible by conventional means.^23^, ^24^, ^25^ This and related methods have been used to measure the microscopic fractional anisotropy without the influence of orientation dispersion in brain,^25^, ^26^, ^27^ brain tumors,^28^ and multiple sclerosis lesions,^29^, ^30^ among others.

Recently, a design scheme for motion‐compensated waveforms that yield tensor‐valued diffusion encoding were proposed by Lasič et al,^31^ thereby extending the range of organs that could be probed by such methods. The waveform design was based on an elegant principle of symmetry^32^ and provides a robust tool for waveform generation. However, the design cannot exploit asymmetric encoding times, only provides compensation for concomitant gradients for a small set of rotations of the waveform, and has suboptimal encoding efficiency.

In this work, we aim to develop a numerically optimized gradient waveform design for tensor‐valued diffusion encoding that uses asymmetric timing with nulling of arbitrary moments of motion as well as concomitant gradients. The proposed design has a superior encoding efficiency and suppresses concomitant gradient effects throughout the imaging volume for arbitrary rotations of the waveform. We also formulate a signal representation that is generalized to tensor‐valued diffusion and motion encoding. Finally, we demonstrate the technical feasibility of several optimized waveforms in the challenging application of in vivo cardiac tensor‐valued diffusion MRI.

## 2 THEORY

Diffusion MR uses magnetic field gradients to encode for the incoherent motion of an ensemble of MR‐visible particles, or “spins.” The signal from an ensemble of spins is the average over all complex spin vectors

(1) $$S=S_{0}\left\langle \exp\left(‐i\phi \right)\right\rangle ,$$

where $S_{0}$ is the relaxation weighted baseline signal; ϕ is the phase of each spin packet; and ⟨·⟩ is the average operator across the ensemble. In the presence of a magnetic field gradient ($g\left(t\right)$), the time‐dependent position of spin ($r\left(t\right)$) is encoded in the phase

(2) $$\phi =\gamma \int_{0}^{\tau }g\left(t\right)r\left(t\right)dt,$$

where tis the time since excitation; τis the TE; and γis the gyromagnetic ratio.^33^ In this paper, we take $g\left(t\right)$ to be the effective gradient (ie, including effects of refocusing pulses). A coherent shift of positions, or bulk motion, will result in a global phase shift, whereas incoherent movement reduces the phase coherence and signal magnitude.^11^, ^34^ To exemplify the principle, we may approximate the signal by using the cumulant expansion,^35^, ^36^ such that $S\approx S_{0}\exp\left(‐\left\langle \phi^{2}\right\rangle /2\right)$. For simplicity, we have assumed that there is no bulk flow (⟨ϕ⟩=0), such that the first nonzero term is the second cumulant (ie, the variance of phases). The phase variance can be decomposed into effects of diffusion (including pseudo diffusion) and ballistic motion (constant direction during the observation time), according to $\left\langle \phi^{2}\right\rangle ={\left\langle \phi^{2}\right\rangle }_{\mathrm{diff}}+{\left\langle \phi^{2}\right\rangle }_{\mathrm{bal}}$. In analogy to anisotropic Gaussian diffusion, described by a diffusion tensor^37^ (D), we may capture the *nth* moment of ballistic flow with a covariance tensor, according to $F_{n}=〈f_{n}^{⊗2}〉‐{〈f_{n}〉}^{⊗2}$. For example, $f_{1}$ is a distribution of velocity vectors, such that multiplication with time gives a distribution of position vectors, given negligible contribution from higher order moments. If the distributions of moment vectors are uncorrelated, normally distributed with zero mean ($\langle f_{n}\rangle =0$), the diffusion and motion‐weighted signal can then be written as

(3) $$S=S_{0}\exp\left(‐B:D‐\sum_{n}\frac{M_{n}:F_{n}}{2{\left(n!\right)}^{2}}\right),$$

where $B=\int_{0}^{\tau }q{\left(t\right)}^{⊗2}dt$ is the b‐tensor^21^;$q\left(t\right)=\gamma \int_{0}^{t}g\left(t^{\prime }\right)dt^{\prime }$ is the dephasing vector; $M_{n}=m_{n}^{⊗2}$ is the rank‐1 motion encoding tensor; “⊗2” denotes the vector outer product; and “:” denotes the double inner product. The motion‐encoding moment of *n*th order is a vector ($m_{n}$), defined from the gradient waveform according to

(4) $$m_{n}=\gamma \int_{0}^{\tau }g\left(t\right)t^{n}dt,$$

where γ is the gyromagnetic ratio; τ is the TE; and *t* is the time from excitation. We assume that the zeroth‐moment vector is always designed to be zero to satisfy the spin‐echo condition, whereas nonzero values for $m_{n}$ encode the velocity (*n* = 1), acceleration (*n* = 2), jerk (*n* = 3), snap (*n* = 4), and so on. To our knowledge, the formalism in Equation 3 is novel and motivated by the use of gradient waveforms that are not colinear in time (ie, yield high‐rank b‐tensors). For example, when $\mathrm{rank}\left(B\right)>1$, the direction of a given motion‐encoding vector must not coincide with other orders of motion encoding, or any b‐tensor eigenvectors. Therefore, we must track each $m_{n}$ as a vector, rather than just a magnitude. Furthermore, the assumptions that $f_{n}$ is normally distributed may not hold in general, reducing the accuracy of Equation 3. Nevertheless, the effects of incoherent motion in the ballistic regime will be suppressed by gradient waveforms with vanishing motion encoding. For example, given sufficient $m_{1}$‐nulling, the phase contribution caused by movement at constant velocity is zero ($\phi_{\mathrm{velocity}}=m_{1}^{}\cdot v\approx 0,\mathrm{because}m_{1}\approx 0$), regardless of the actual distribution of velocities.

## 3 METHODS

### 3.1 Numerical optimization of motion‐compensated tensor‐valued diffusion encoding

We generate motion‐compensated gradient waveforms for tensor‐valued diffusion encoding by extending the numerical optimization framework by Sjölund et al^38^ to include constraints on motion encoding. This is in addition to the original constraints that can be applied to the zeroth moment ($m_{0}$), b‐tensor shape, gradient amplitude, slew rate, and heat dissipation,^38^ as well as compensation of concomitant gradients.^39^ Because the motion encoding is treated as a vector ($\mathrm{rank}(M_{n})\leq 1$), we may impose a nonlinear optimization constraint on the magnitude of the *n*th‐moment vector to an arbitrary magnitude threshold ($L_{n}$), such that

(5) $$\left|m_{n}\right|\leq L_{n},$$

or impose a linear equality constraint

(6) $$m_{n}=0.$$

Although both methods were implemented in the optimization framework, the type and limits on motion encoding should be adapted to the intended use case. For example, the linear constraint in Equation 6 results in faster optimization and is useful for removing motion encoding entirely (nulled to within numerical precision), whereas Equation 5 facilitates motion encoding of a specified value, which allows a larger solution space and may be beneficial with respect to encoding efficiency. For the purposes of a general demonstration, we used relatively restrictive thresholds on motion encoding, such that $L_{0}$ = 0, $L_{1}$ = 10^−4^, $L_{2}$ = 10^−4^ in units of s*^n^*/m, assuming that $\gamma =2.675\cdot 10^{8}$ rad/s/T for hydrogen in Equation 4. For comparison, $\left|m_{1}\right|$ and $\left|m_{2}\right|$ for the noncompensated monopolar waveforms, optimized for the same imaging conditions, are approximately eight orders of magnitude larger. We adopt the convention that “**m** *_n_*‐nulling” means constraining the magnitude of all moment vectors up to, and including, the *n*th order.

Throughout the optimization, we also constrained the maximal gradient amplitude to 80 mT/m, the maximal slew rate to 60 T/m/s, without additional constraint on heat dissipation.^38^ Waveforms were optimized for linear, planar, and spherical b‐tensor encoding using both max‐norm and L2‐norm constraints. Briefly, the max‐norm means that $g\left(t\right)$ is inscribed within a cube that is 160 mT/m on each side, whereas the L2‐norm limits $g\left(t\right)$ within a sphere with diameter 160 mT/m, the latter being less efficient but can be arbitrarily rotated without exceeding the maximal gradient amplitude.^22^ All variants were compensated for concomitant gradients by “M‐nulling,”^39^ whereby the Maxwell index was limited to 100 (mT/m)^2^ ms. Additionally, a variant for spherical b‐tensor encoding was optimized using “K‐nulling” to be more comparable to the design proposed by Lasič et al.^31^ Briefly, K‐nulling yields slightly higher encoding efficiency, but unlike M‐nulling, the waveforms are not compensated for concomitant gradients when rotated^39^ and/or affected by gradient nonlinearity.^40^ The duration of the waveform was minimized under the requirement that it yield a b‐value of 2 ms/µm^2^, assuming a spin‐echo sequence with a timing asymmetry such that the encoding period before the refocusing pulse was 3 ms longer than the period after, and in which the refocusing required 8 ms.

### 3.2 Evaluation of waveform efficiency and simulation of motion compensation

The efficiency of waveforms with variable limits on motion encoding was investigated in terms of the necessary encoding time to reach *b* = 2 ms/μm^2^ as well as the encoding efficiency factor (κ)^38^, ^41^

(7) $$\kappa =\frac{4b}{\gamma^{2}g_{\text{max}}^{2}t_{\text{tot}}^{3}},$$

where the achievable b‐value is related to the maximal gradient amplitude per axis $g_{\text{max}}$ and the total duration of the diffusion encoding gradient waveform $t_{\text{tot}}$. For completeness, we also describe the efficiency for nulling up to $m_{6}$. This is performed for symmetric encoding times, such that periods available for diffusion encoding before and after the refocusing are equal (δ_1_ = δ_2_ = 30 ms), and a realistic^42^ asymmetric timing (δ_1_ = 33 ms, δ_2_ = 27 ms). In both cases, periods are separated by 8 ms to accommodate the refocusing block.

We perform numerical simulations to explore the conditions under which the generated waveforms are compensated for motion when they are scaled to yield *b* = 2 ms/μm^2^. We simulate the signal from 10^5^ spins according to Equations 1 and 2, where the time‐dependent position is $r\left(t\right)=\sum_{n=0}f_{n}t^{n}/n!=r_{0}+vt+at^{2}/2+jt^{3}/6+st^{4}/24+\cdots$. The initial position ($r_{0}$) can be set to zero, and values for all other moments are defined at t=0. Each kind of motion is assumed to be normally distributed with zero mean and a given SD, such that the elements of the motion moment vector $f_{n}={\left[f_{n,x}f_{n,y}f_{n,z}\right]}^{T}$are independently sampled from a normal distribution $f_{n}\in N\left(0,\sigma_{f_{n}}^{2}\right)$. During tests of the efficacy of $m_{1}$ and $m_{2}$‐nulling, the maximal values for the SDs were $\sigma_{v}=10$ m/s and $\sigma_{a}=$ 100 m/s^2^, while higher‐order terms were equal to zero. These limits are orders of magnitude greater than the motion observed for in vivo cardiac imaging,^43^, ^44^ and should therefore cover the worst‐case scenario. Furthermore, we gauge the sensitivity to higher‐order moments of motion that are not explicitly accounted for in the optimization. To this end, we estimate the SD of “jerk” and “snap” ($\sigma_{j},\sigma_{s}$) required to reduce the signal by approximately 1% for waveforms that were $m_{2}$‐nulled.

### 3.3 Optimized waveforms in cardiac imaging

To demonstrate the technical feasibility of this approach, we deployed motion‐compensated waveforms in a healthy heart in vivo. The heart provides a challenging testbed where the effects of motion and motion compensation can be easily appreciated. Data acquisition was conducted in accordance with the Declaration of Helsinki and was approved by the UK National Research Ethics Service (18/YH/0168). The volunteer provided written, informed consent.

Images were acquired on a 3T Prisma (Siemens Healthcare, Erlangen, Germany) with a prototype spin‐echo sequence^42^ using linear and planar b‐tensors with $m_{0}$, $m_{1}$ and $m_{2}$‐nulling. We used these two b‐tensor shapes for their superior encoding efficiency^22^, ^38^ and because they are likely to contribute the most information in the context of microstructure imaging.^45^ We used a reduced FOV echo‐planar readout (ZOOMit^46^), TR = 5 RR‐intervals, TE = [77, 93, 99] ms, partial Fourier = 6/8, resolution = 3 × 3 × 8 mm^3^, five slices, in‐plane FOV = 320 × 118 mm^2^, slice gap = 8 mm, and *b* = [0.1, 0.4, 0.7, 1.0] ms/μm^2^ in 6, 6, 15, and 30 rotations, respectively (single repetition). At the highest b‐value, the maximal per‐axis gradient amplitude was 78.6 mT/m (Supporting Information Figure S1). The rotations aligned the symmetry axis of the b‐tensors along direction sets based on platonic solids.^21^, ^47^ The acquisition was cardiac‐triggered under free breathing, and images were acquired in midsystole. To maintain consistency in cardiac phase, while accounting for different diffusion waveform durations and TE, the trigger delay was set to approximately 50% of time to maximum systole minus TE. Finally, we note that the optimized gradient waveforms had large enough zeroth moments after the refocusing pulse to act as crushers even at the lowest b‐value (all above 3∙10^4^ m^−1^). Therefore, crushers were never engaged and did not contribute to motion encoding.

In addition to diffusion‐weighted images, we visualize signal dropout in terms of the initial slope of log(*S*) versus *b*, or mean diffusivity (MD=Trace(D)/3), where D is estimated from a diffusion tensor representation (Equation 3 without influence from motion). In this context, we do not use MD to evaluate microstructure; rather, high MD values are used to detect strong signal dropout as a function of encoding strength, which indicates poor motion compensation. The fitting was adapted for tensor‐valued diffusion encoding and used the open‐source multidimensional diffusion MRI framework^48^ (https://github.com/markus‐nilsson/md‐dmri).

## 4 RESULTS

**FIGURE 1:**
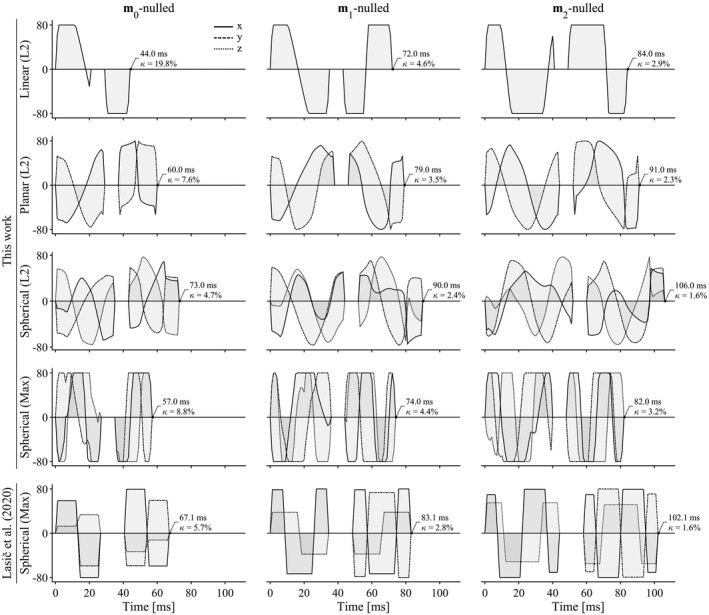
Gradient waveforms generated by the proposed design (top four rows) and the design by Lasic et al^5^ for reference (bottom row). The duration of all waveforms is minimized under the condition that they produce *b* = 2 ms/µm^2^ and spherical b‐tensors, at maximal gradient amplitude of 80 mT/m, maximal slew rate of 60 T/m/s, in a spin‐echo sequence in which the refocusing requires 8 ms, and the first encoding period is 6 ms longer than the second. The waveforms from this work use M‐nulling for compensating concomitant gradients, whereas the bottom row uses K‐nulling.^39^ The notation in parenthesis denotes waveforms constrained within a sphere (L2‐norm) or a cube (Max‐norm).^38^ The proposed method for **m**_1_ and **m**_2_‐nulling is more efficient than that by Lasic et al,^5^ as evidenced by the 10‐20 ms reduction to encoding time (compare bottom two rows using similar optimization constraints)

**FIGURE 2:**
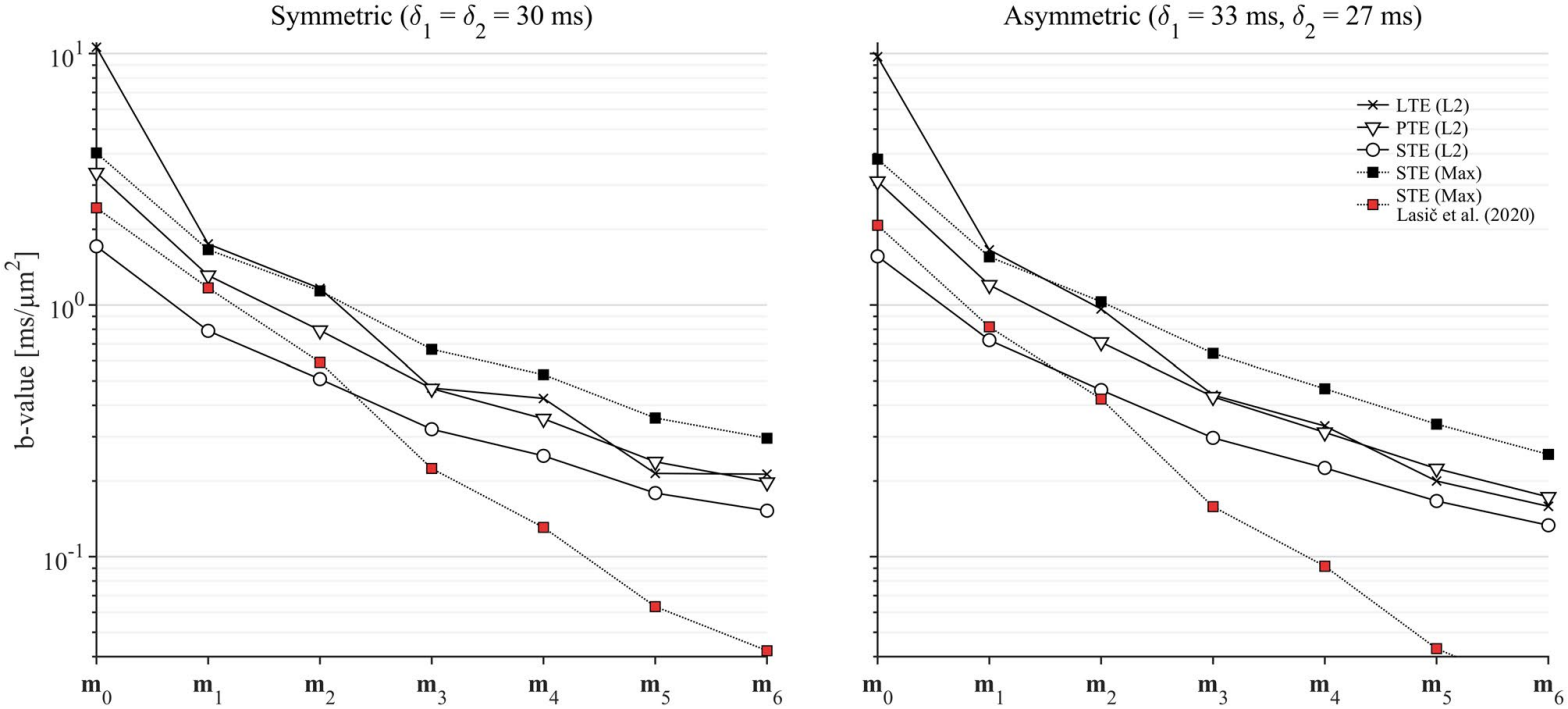
The achievable b‐value is reduced as higher moments of the waveform are constrained. Plots show b‐values for symmetric and asymmetric encoding periods, both separated by 8 ms (for refocusing), as a function of the order of motion compensation from **m**_0_ to **m**_6_, corresponding to position, velocity, acceleration, jerk, snap, crackle, and pop. The proposed design yields superior encoding efficiency compared with Lasic et al^5^ (compare Max‐norm waveforms with square markers), especially for increasing timing asymmetry and order of moment that is nulled. Note that for a symmetric timing and linear b‐tensor encoding, the efficiency does not strictly decrease with the nulled moment order. Abbreviations: LTE, linear b‐tensor encoding; PTE, planar b‐tensor encoding; STE, spherical b‐tensor encoding

The optimization framework robustly generated waveforms that fulfilled all optimization criteria for arbitrary shapes of the b‐tensor. Figure 1 shows examples of waveforms that yield *b* = 2 ms/µm^2^ for varying b‐tensor shapes and levels of motion compensation. As expected, the efficiency generally deteriorates as higher moments are nulled, as indicated by longer encoding times, and lower efficiency factors. We also observed that K‐nulling yields a slight efficiency advantage over M‐nulling (Supporting Information Figure S2). Figure 2 shows the achievable b‐values for symmetric and asymmetric timing up to $m_{6}$‐nulling. Again, constraints on ever higher moments reduce the encoding efficiency. The proposed design is more efficient than that by Lasic et al,^5^ reducing the necessary encoding time for **m**_1_ and **m**_2_‐nulled waveforms by 9‐20 ms, and the benefit of the current design increases dramatically with increasing order of nulling. Our design can also yield waveforms for linear b‐tensor encoding without compensation for concomitant gradients that match the efficiency of the framework by Aliotta et al,^16^ and with M‐nulling it yields results similar to those by Peña‐Nogales et al^17^ (Supporting Information Figure S3). Somewhat unexpectedly, we observe that waveforms for linear b‐tensor encoding and symmetric timing can be more efficient when nulling even moments compared with preceding odd moments (see Figure 2 where **m**_6_‐nulling is more efficient than **m**_5_‐nulling), which indicates that the global minimum was not found. We observed that the combination of linear b‐tensors, symmetric timing, and nulling of odd moments tends to produce self‐balanced waveforms (q(τ/2)=0) that do not exert diffusion encoding during the refocusing period, and therefore suffer a loss to efficiency.

**FIGURE 3:**
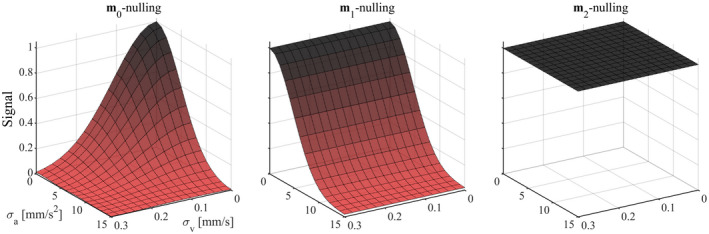
The surfaces show signal attenuation as a function of velocity and acceleration distributions for waveforms that are nulled for different orders of motion encoding. The simulation assumed ballistic motion with normal velocity and acceleration distributions with SDs in the intervals $\sigma_{v}\in \left[0,0.3\right]$ mm/s and $\sigma_{a}\in \left[0,15\right]$ mm/s^2^. All waveform designs exhibited similar behavior: The **m**_0_‐nulled waveforms were sensitive to velocity and acceleration (left); **m**_1_‐nulling removed the effects of velocity but not acceleration (middle); and **m**_2_‐nulling made the measurement insensitive to both velocity and acceleration (right). The relatively narrow intervals for $\sigma_{v}$ and $\sigma_{a}$ are selected to show regions where the signal is dynamic. However, we note that **m**_1_‐nulling was invariant to velocity up to at least $\sigma_{v}=10$ m/s, and **m**_2_‐nulled waveforms are invariant to acceleration up to at least $\sigma_{a}=100$ m/s^2^

Numerical simulations in Figure 3 show signal dephasing due to motion for different levels of motion compensation. In general, noncompensated waveforms suffer a gross loss of signal due to all kinds of incoherent motion; **m**_1_‐nulling removes the effects of velocity, whereas **m**_2_‐nulling removes the effects of both velocity and acceleration, as intended by the design. Note that the intervals of $\sigma_{v}$ and $\sigma_{a}$ depicted in Figure 3 are relatively narrow to show the regions where signal is dynamic, but that waveforms nulled for **m**_1_ were invariant to velocity, and **m**_2_‐nulled waveforms were invariant to velocity and acceleration throughout the entire simulated interval. For reference, we report that the resulting magnitude of the jerk and snap‐encoding vectors for **m**_2_‐nulled waveforms were |**m**_3_| = 56‐300 m/s^3^ and |**m**_4_| = 1.5‐14 m/s^4^. Both numerical simulations and Equation 3 show that this leads to a loss of approximately 1% of signal for distributions where $\sigma_{j}=50$ mm/s^3^ and $\sigma_{s}=1$ m/s^4^.

**FIGURE 4:**
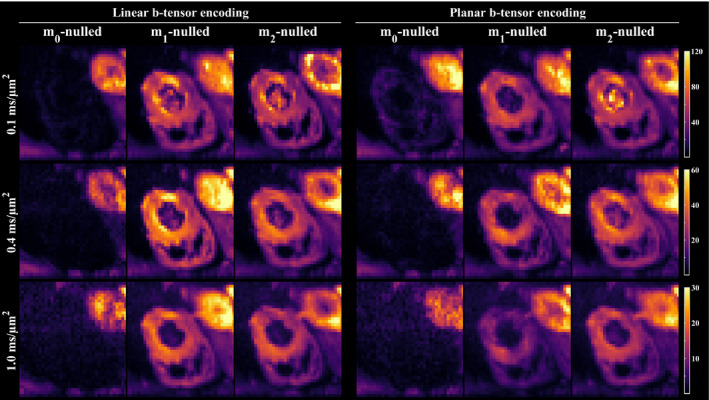
Signal maps in the mid‐myocardium encoded by linear and planar b‐tensors, averaged over directions, show that motion compensation improves the data quality. Noncompensated waveforms suffer gross signal loss due to motion, whereas the proposed method for nulling **m**_1_ and **m**_2_ retains the signal even at relatively high b‐values

**FIGURE 5:**
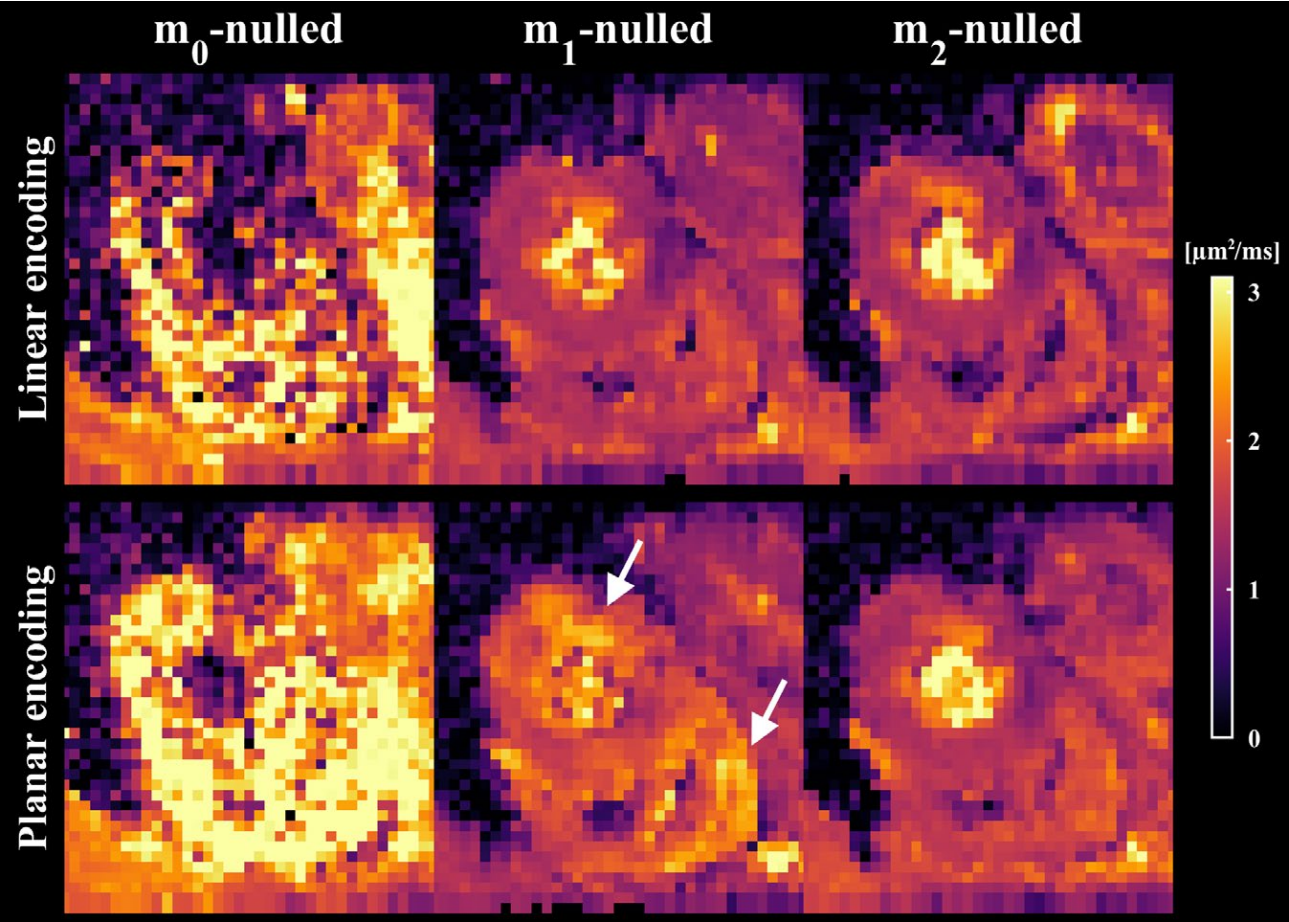
Maps of mean diffusivity show that waveforms that are not compensated for motion cannot be used reliably for in vivo cardiac imaging. Waveforms that are **m**_1_‐nulled (velocity compensation) result in a marked improvement in image quality; however, some regions still exhibit elevated mean diffusivity, whereby artifacts cannot be ruled out (white arrows). As expected, **m**_2_‐nulled (velocity and acceleration compensation) waveforms appear the most robust. Maps from multiple slices are available in the Supporting Information

Figure 4 shows a single slice of the signal maps averaged over directions in the cardiac muscle when using different levels of motion compensation and b‐tensor shapes. As expected, there is marked loss of signal in the myocardium for all noncompensated waveforms, regardless of b‐value. In contrast, **m**_1_ and **m**_2_‐nulled waveforms consistently retain signal and provide a marked improvement on the data quality. The improved data quality of motion‐compensated waveforms can also be appreciated in the MD maps, where signal dropout is indicated by high MD values in Figure 5. Although some regions were still hyperintense for **m**_1_‐nulled waveforms, **m**_2_‐nulling appeared homogeneous throughout the cardiac muscle for linear and planar b‐tensor encoding (Supporting Information Figure S4). Although the motion‐compensated waveforms were successfully executed, it remains difficult to distinguish features caused by artifacts related to cardiac imaging versus motion compensation.

## 5 DISCUSSION AND CONCLUSIONS

We have proposed and demonstrated a novel design for motion‐compensated gradient waveforms for tensor‐valued diffusion encoding. The suggested design has several benefits over previous designs: (1) higher encoding efficiency than previous designs for tensor‐valued diffusion encoding; (2) a flexible optimization framework that leverages asymmetric waveform timing with arbitrary distribution of the encoding time, user defined energy consumption, and level of motion compensation; and (3) compensation for concomitant gradient effects by M‐nulling, which allows for arbitrary rotations^39^ and is robust to gradient nonlinearity.^40^ We emphasize that compensating for concomitant gradients should not be overlooked, especially in body imaging in which FOVs and voxels are relatively large, and the target tissue may be far from the isocenter.^49^, ^50^ Concomitant gradients can also be suppressed by estimating them in a point in space and subtracting them from the desired gradient waveform during execution of the pulse sequence.^16^, ^50^ However, the benefit of the current method is that concomitant gradient effects are removed throughout the entire imaging volume, rather than one point at a time, and therefore is more compatible with large FOVs and multislice imaging. We also note that the current design does not force “self‐balanced” waveforms ($q\left(t\right)$ is not zero during the refocusing pulse), which provides improved efficiency and may remove the need/influence of additional crusher gradients that disturb the motion compensation. Consider that the motion encoding of a pair of crushers is on the order of |**m**_1_| = 10‐100 s/m and |**m**_2_| = 1‐10 s^2^/m. However, the inclusion of crushing is currently not enforced by the optimization, and an investigation of the effects of various pulse‐sequence configurations and imaging gradients on the motion compensation was outside the scope of this study.

We expect that this waveform design will improve the feasibility and quality of microstructure imaging that relies on tensor‐valued encoding in organs that require special attention to ballistic motion, such as cardiac, liver, and kidney imaging.^5^, ^6^, ^12^, ^13^, ^51^ In this work, we explored the numerical effects of flow and acceleration over a wide range of values, and established the magnitude at which the higher moments, jerk, and snap become relevant. However, it remains the responsibility of the user to determine the appropriate level and order of motion compensation to use in gradient waveform optimization, and to account for organ‐specific challenges in the remainder of the experimental design.

The design presented herein can also be extended to incorporate effects of diffusion time and exchange by using similar constraints on related metrics.^22^, ^52^, ^53^, ^54^ Doing so allows us to emphasize or suppress diffusion time and exchange effects, which may otherwise confound the measurement.^22^, ^31^, ^55^, ^56^, ^57^, ^58^ Furthermore, the current design can already produce waveforms with independently controlled motion and diffusion sensitization, facilitating an interesting probe of diffusion‐motion‐correlation experiments. Such multidimensional experiments will be explored in future studies.

## CONFLICT OF INTEREST

F.S. is an inventor on patents related to this study. The remaining authors declare no conflict of interest.

## Supporting information


**FIGURE S1** Gradient waveforms used in the in vivo experiments nulled for **m**_0_, **m**_1_, and **m**_2_. Each plot shows the maximal gradient amplitude exerted on any one axis, and the duration of encoding before (*δ*_1_) and after (*δ*_2_) the refocusing. For each order of nulling, the sequence timing for linear and planar b‐tensors was the same due to the fixed TE
**FIGURE S2** Gradient waveforms with **m**_0_, **m**_1_, and **m**_2_‐nulling from spherical b‐tensor encoding, in which the optimization in the top row used K‐nulling, and the bottom row used M‐nulling.^39^ The title of each plot shows the achieved b‐value for the given timing. M‐nulling is generally somewhat less efficient, but more versatile, as it allows arbitrary rotations of the waveform and is robust to gradient nonlinearity^39^, ^40^
**FIGURE S3** Comparison of waveforms for linear b‐tensor encoding, nulled for moments up to **m**_2_ using optimization frameworks of the present work^38^, ^39^ (NOW, https://github.com/jsjol/NOW), by Aliotta et al.^16^ (CODE, https://github.com/ealiotta/code‐gradient‐design), and by Peña‐Nogales et al^17^ (ODGD, https://github.com/opennog/ODGD). “MX” in the name indicates that the waveform is compensated for concomitant gradient effects. Overall, the different frameworks yield similar results. The case in the lower right is an outlier, likely due to an incorrect derating of the gradient. Red lines show the evolution of the motion‐encoding vectors, scaled to arbitrary units for visibility
**FIGURE S4** Maps of mean diffusivity (MD) indicate regions of signal dropout in multiple slices in a healthy heart. Although **m**_1_‐nulling provides a vast improvement in data quality over **m**_0_, some hyperintense regions remain, seen especially for planar b‐tensor encoding. However, **m**_2_‐nulling appears to yield high data quality throughout the heart muscle

## Data Availability

The numerical optimization framework, including the proposed motion compensation, is made available in open source at https://github.com/jsjol/NOW, commit 354cfb3. All gradient waveforms mentioned in this work, signal sampling schemes, and related resources are shared at https://github.com/filip‐szczepankiewicz/Szczepankiewicz_MRM_2020, commit ce42e91.
