# Supplementary material for: Motion‐compensated gradient waveforms for tensor‐valued diffusion encoding by constrained numerical optimization
Source: Magn Reson Med. 2020 Oct 13;85(4):2117–26. doi: 10.1002/mrm.28551 (PMC7821235; doi:10.1002/mrm.28551)
Supplement: Supplementary file 1 — FIGURE S1 Gradient waveforms used in the in vivo experiments nulled for m 0, m 1, and m 2. Each plot shows the maximal gradient amplitude exerted on any one axis, and the duration of encoding before (δ 1) and after (δ 2) the refocusing. For each order of nulling, the sequence timing for linear and planar b‐tensors was the same due to the fixed TE FIGURE S2 Gradient waveforms with m 0, m 1, and m 2‐nulling from spherical b‐tensor encoding, in which the optimization in the top row used K‐nulling, and the bottom row used M‐nulling. 39 The title of each plot shows the achieved b‐value for the given timing. M‐nulling is generally somewhat less efficient, but more versatile, as it allows arbitrary rotations of the waveform and is robust to gradient nonlinearity 39 , 40 FIGURE S3 Comparison of waveforms for linear b‐tensor encoding, nulled for moments up to m 2 using optimization frameworks of the present work 38 , 39 (NOW, https://github.com/jsjol/NOW), by Aliotta et al. 16 (CODE, https://github.com/ealiotta/code‐gradient‐design), and by Peña‐Nogales et al 17 (ODGD, https://github.com/opennog/ODGD). “MX” in the name indicates that the waveform is compensated for concomitant gradient effects. Overall, the different frameworks yield similar results. The case in the lower right is an outlier, likely due to an incorrect derating of the gradient. Red lines show the evolution of the motion‐encoding vectors, scaled to arbitrary units for visibility FIGURE S4 Maps of mean diffusivity (MD) indicate regions of signal dropout in multiple slices in a healthy heart. Although m 1‐nulling provides a vast improvement in data quality over m 0, some hyperintense regions remain, seen especially for planar b‐tensor encoding. However, m 2‐nulling appears to yield high data quality throughout the heart muscle [file MRM-85-2117-s001.docx]

**Supporting Information**





Figure S1 – Gradient waveforms used in the in vivo experiments nulled for **m­**_0_, **m**_1_, and **m**_2_. Each plot shows the maximal gradient amplitude exerted on any one axis, and the duration of encoding before (*δ­*_1_) and after (*δ­*_2_) the refocusing. For each order of nulling, the sequence timing for linear and planar b-tensors was the same due to the fixed echo time.





Figure S2 – Gradient waveforms with **m­**_0_, **m**_1_ and **m**_2_-nulling from spherical b-tensor encoding where the optimization in the top row used K-nulling, and the bottom row used M-nulling.^1^ The title of each plot shows the achieved b-value for the given timing. M-nulling is generally somewhat less efficient, but more versatile since it allows arbitrary rotations of the waveform and is robust to gradient nonlinearity.^1, 2^


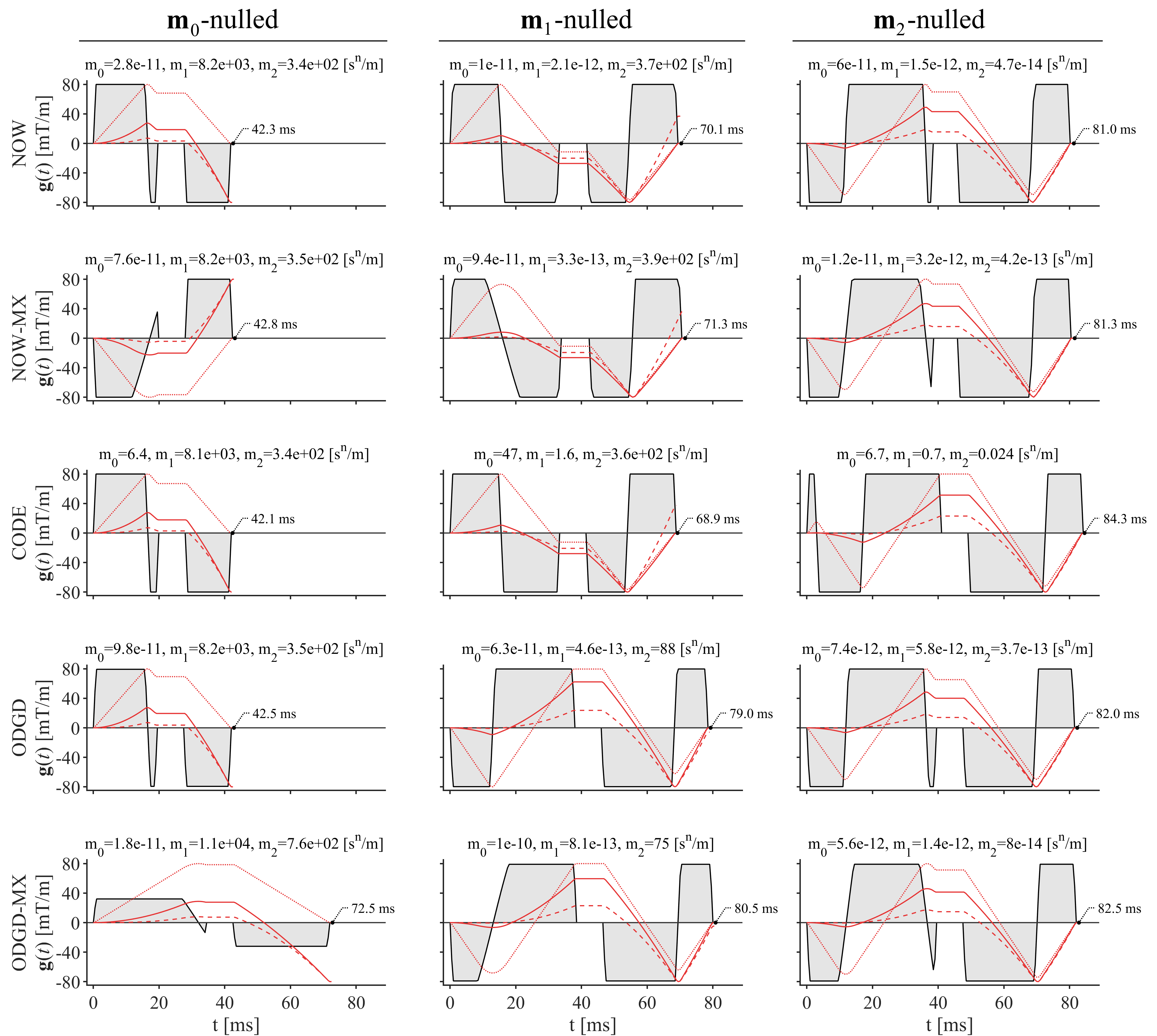


Figure S3 – Comparison of waveforms for linear b-tensor encoding, nulled for moments up to **m**­_2_ using optimization frameworks of the present work^1, 3^ (NOW, <https://github.com/jsjol/NOW>), by Aliotta et al.^4^ (CODE, <https://github.com/ealiotta/code-gradient-design>), and by Peña-Nogales et al.^5^ (ODGD, <https://github.com/opennog/ODGD>). The ‘MX’ in the name indicates that the waveform is compensated for concomitant gradient effects. Overall, the different frameworks yield similar results. The case in the lower right is an outlier, likely due to an incorrect derating of the gradient. Red lines show the evolution of the motion encoding vectors, scaled to arbitrary units for visibility.


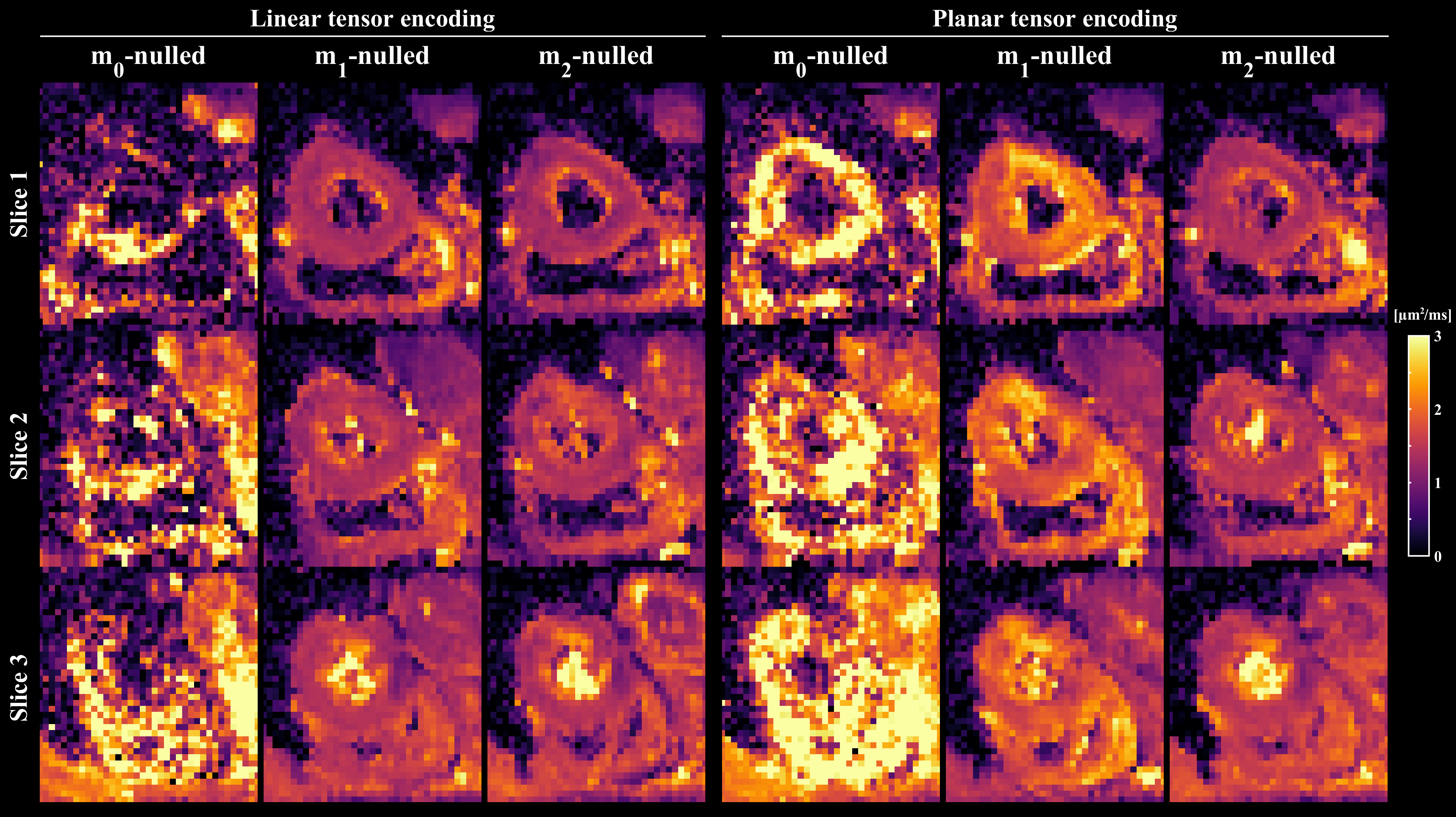


Figure S4 – Maps of mean diffusivity (MD) indicate regions of signal dropout in multiple slices in a healthy heart. Although **m**_1_-nulling provides a vast improvement in data quality over **m**_0_, some hyperintense regions remain, seen especially for planar b-tensor encoding. However, **m**_2_-nulling appears to yield high data quality throughout the heart muscle.

**References**

1. Szczepankiewicz F, Westin CF, Nilsson M. Maxwell-compensated design of asymmetric gradient waveforms for tensor-valued diffusion encoding. *Magn Reson Med*. May 31 2019;doi:10.1002/mrm.27828

2. Szczepankiewicz F, Eichner C, Anwander A, Westin C-F, Paquette M. The impact of gradient non-linearity on Maxwell compensation when using asymmetric gradient waveforms for tensor-valued diffusion encoding. 2020:

3. Sjölund J, Szczepankiewicz F, Nilsson M, Topgaard D, Westin CF, Knutsson H. Constrained optimization of gradient waveforms for generalized diffusion encoding. *J Magn Reson*. Oct 31 2015;261:157-168. doi:10.1016/j.jmr.2015.10.012

4. Aliotta E, Wu HH, Ennis DB. Convex optimized diffusion encoding (CODE) gradient waveforms for minimum echo time and bulk motion-compensated diffusion-weighted MRI. *Magn Reson Med*. Feb 2017;77(2):717-729. doi:10.1002/mrm.26166

5. Peña-Nogales Ó, Zhang Y, Wang X, de Luis-Garcia R, Aja-Fernandez S, Holmes JH, Hernando D. Optimized Diffusion-Weighting Gradient Waveform Design (ODGD) formulation for motion compensation and concomitant gradient nulling. *Magn Reson Med*. Feb 2019;81(2):989-1003. doi:10.1002/mrm.27462
